# Supplementary material for: Development of a Targeted Choroidal Injury Model for the Study of Retinal Degenerations and Therapeutic Cell Replacement
Source: Transl Vis Sci Technol. 2026 Aug 20;15(8):17. doi: 10.1167/tvst.15.8.17 (PMC13499576; doi:10.1167/tvst.15.8.17)
Supplement: Supplementary file 1 [file tvst-15-8-17_s001.pdf]

Supplementary Information

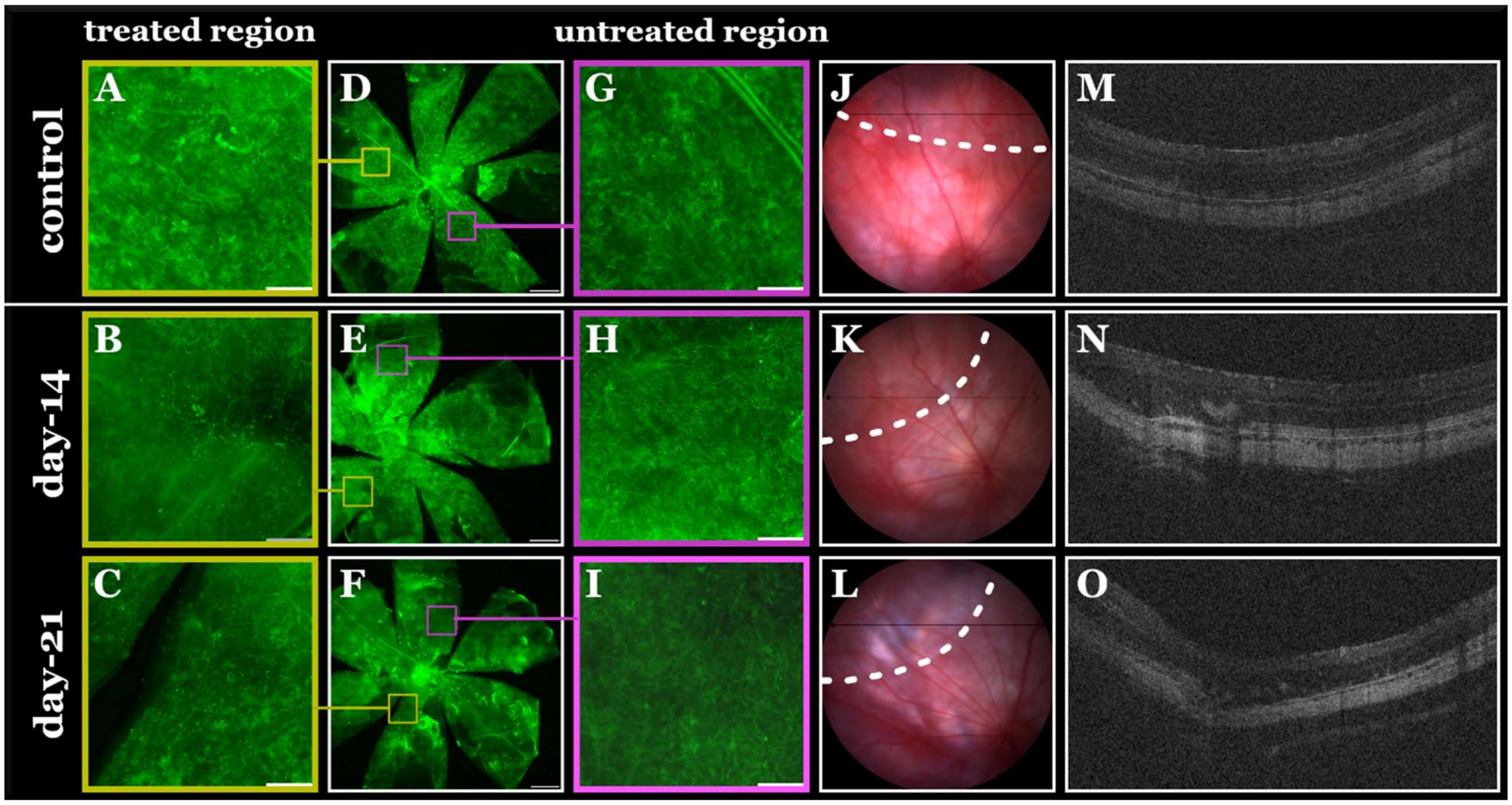

Supplementary Figure 1. Suprachoroidal anti-CD105-SAP injection causes sectoral choroidal and outer retinal loss. Suprachoroidal injection of anti-CD105-SAP (0.5 µg/eye) causes sectoral choroidal and outer retinal loss compared to eyes injected with control (PBS only; top row). High magnification inserts (1st and 3rd column; scale bar 100 µm) from flat mounts (D-F; Scale bar is 1000 µm) demonstrate focal choroidal injury in treated areas (A-C, Scale bar is 100 µm) and preserved choroidal vasculature in untreated areas (G-I, Scale bar is 100 µm). Corresponding fundus photographs, with the site of injection, showing in white dashed lines(J-L) and OCT line scans (M-O) show sectoral regions of choroidal and outer retinal damage, which persists through all studied time points.

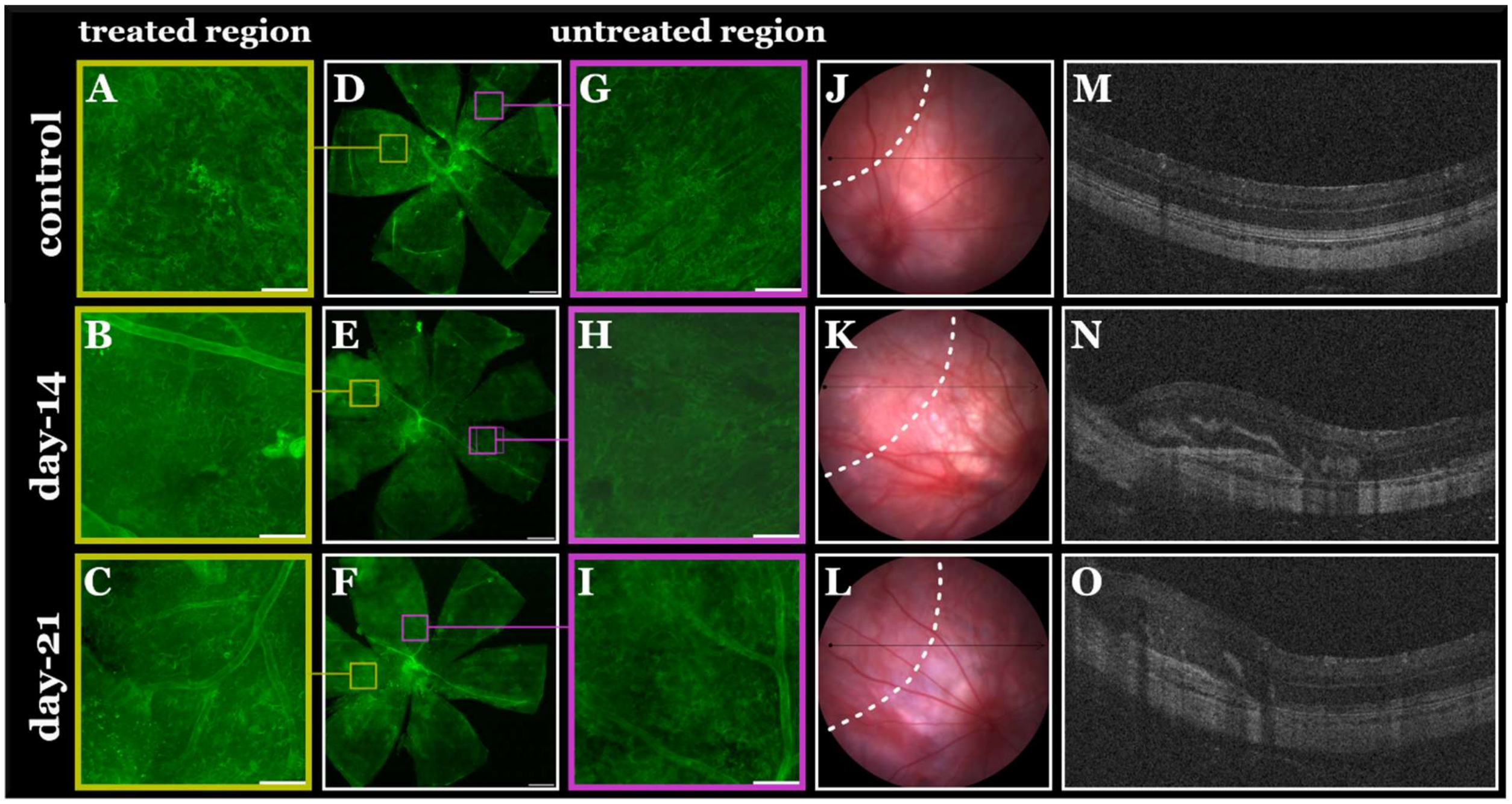

Supplementary Figure 2. Suprachoroidal anti-CD38-SAP injection causes sectoral choroidal and outer retinal loss. CD38-SAP suprachoroidal injection leads to sectoral loss of choroidal and outer retinal tissue compared to eyes that received the control injection (PBS only; top row). High magnification images (1st and 3rd column; scale bar 100 µm) from choroid scleral flat mounts reveal localized choroidal damage in the treated regions (A-C, Scale bar is 100 µm), while the choroidal vasculature remains intact in untreated areas (G-I, scale bar is 100 µm). Corresponding fundus images, with the site of injection, showing in white dashed lines (J-L) and OCT scans (M-O) display distinct sectors with choroidal and outer retinal damage.

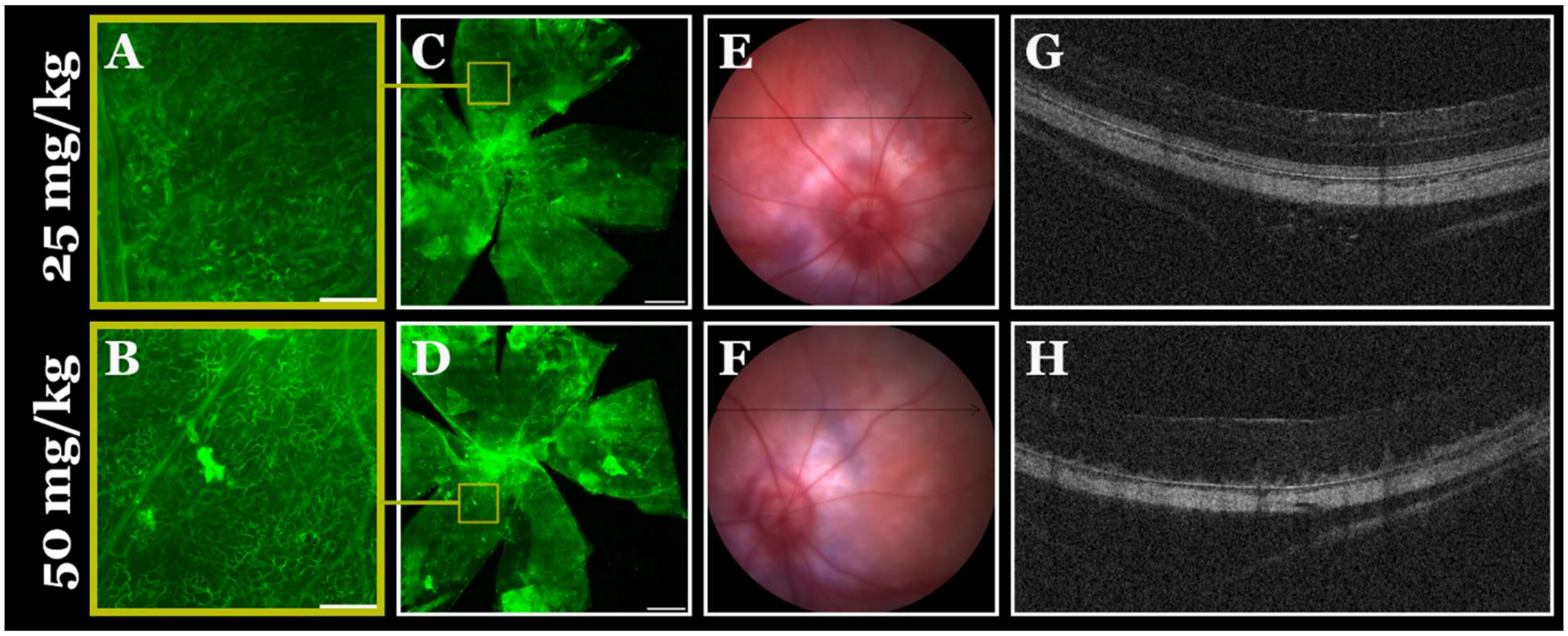

Supplementary Figure 3. Systemic administration of sodium iodate in SRG rats results in dose-related choroidal and outer retinal damage at 1-week post-treatment. Intravenous sodium iodate resulted in diffuse choroidal and outer retinal loss in a dose-related fashion (doses shown on left) by 1 week after treatment. High magnification inserts (A,B; Scale bar is 100 µm) from flat mounts (C,D); Scale bar is 1000 µm) demonstrate choroidal injury. Corresponding fundus photographs (E,F) and OCT line scans (G,H) show diffuse choroidal and outer retinal damage. Note the minimal choroidal and outer retinal damage in low-dose treated animals (25 mg/kg; top row).
